# Jaw lift causes less laryngeal interference during lightwand-guided intubation than combined jaw and tongue traction applied by single operator

**DOI:** 10.4103/0019-5049.79896

**Published:** 2011

**Authors:** Umesh Goneppanavar, Akshay Nair, Gurudas Kini

**Affiliations:** Department of Anaesthesiology, Kasturba Medical College, Manipal, India

**Keywords:** Epiglottis, general anaesthesia, intubation, laryngoscopy, tongue

## Abstract

Lightwand-guided intubation is a semi-blind technique that takes advantage of the anterior location of the trachea in relation to the oesophagus. Fibreoptic evaluation of lightwand-guided intubation has revealed a possibility of laryngeal interference and epiglottic distortion. Jaw lift, tongue traction or a combination of both have been used to assist in lightwand-guided intubation. This study fibreoptically evaluates lightwand-guided intubation using jaw lift and combined jaw and tongue traction. Eighty four patients with normal airway undergoing general anaesthesia were studied. This randomised, double blinded, cross over study was done in two phases. First phase – after achieving adequate depth of anaesthesia, a fibrescope was advanced nasally, and lightwand-guided intubation was carried out under direct fibreoptic visualisation with the aid of either jaw lift or combined jaw and tongue traction. Second phase – Extubation followed by reintubation using the other manoeuvre. Interference with laryngeal structures during intubation and position of the epiglottis at the end of intubation were noted. Epiglottic distortion (deviated to one side/infolded into trachea) was observed in 6 patients with jaw lift and 17 patients with combined jaw and tongue traction (*P*=0.003). Laryngeal interference was significantly higher (*P*=0.012) with combined manoeuvre (30/78) than with jaw lift alone (9/81). Although lightwand-guided intubation can be performed quickly and easily, interference with laryngeal structures and distortion of the epiglottis can occur. Jaw lift manoeuvre causes less laryngeal interference than combined jaw and tongue traction applied by a single operator.

## INTRODUCTION

Transillumination ofthe soft tissues of the neck using a lighted-stylet (lightwand) is one of themany alternative intubating techniques developed during the pastdecades. Intubation using lightwand is a light-guided technique without visualisation of the laryngeal structures.[1] TheTrachlight™ (Laerdal Medical Inc., Armonk, NY), from the family of lightwands, has overcome most of the limitations observed with former devices. Gentleness of its intubation technique is demonstrated by the low incidence of mucosal injury and the absence of dental trauma when compared to laryngoscopy.[2] However, fibreoptic evaluation of lightwand-guided intubation has revealed that the epiglottis sometimes hinders the passage of lightwand, and may also get malpositioned into the laryngeal inlet during the process of intubation.[3] Epiglottic malposition has been also reported to occur with intubation attempts with the use of intubating laryngeal mask airway and fibreoptic bronchoscope.[4,5] Subluxation of the cricoarytenoid cartilage has also been reported in a study using an older version of a lightwand (Tubestat).[6] Hence, it becomes very important that the airway manoeuvres that were once recommended to be applied during difficult lightwand-guided intubation may need to be applied routinely during lightwand-guided intubation to minimise laryngeal injury. Suggested manoeuvres to overcome difficulty in lightwand-guided intubation are jaw lift or tongue traction or a combination of both.[2] No formal study has evaluated routine application of these manoeuvres for minimising the laryngeal interference during lightwand-guided intubation. This study aims to fibreoptically evaluate Trachlight™ guided intubation process while using two of these airway manoeuvres, jaw lift (the most commonly employed manoeuvre during lightwand-guided intubation) and combined jaw and tongue traction (combined manoeuvre).

## METHODS

After obtaining the approval from the Institutional Ethics Committee, a total of 84 patients were included in this study which was prospective, randomised, double blinded and cross-over in design. Written informed consent was obtained from patients of either gender aged above 18 years and belonging to American Society of Anesthesiologists’ Physical Status 1 or 2 scheduled for elective surgery requiring orotracheal intubation. Patients with known or anticipated difficulty in mask ventilation; full stomach; intra-oral/nasal pathology; neck mass or contracture neck/thick neck and temporomandibular ankylosis were excluded from the study.

All patients were reviewed preoperatively and demographics were noted: age (years), gender, and weight (kg) and body mass index. Standard fasting orders were followed. Appropriate premedication was given as deemed necessary. The Trachlight™ (Laerdal Medical Inc., Armonk, NY, USA), a new lightwand device was used to intubate the trachea.

Three observers were involved in the conduct of the study. Observer 1, an anaesthesiologist experienced in fibreoptic bronchoscopy who did fibreoptic assessment of the larynx and the intubation process. Observer 2, an anaesthesiologist experienced in Trachlight™ guided intubations who applied the necessary airway manoeuvres and performed the Trachlight™ guided intubation. Observer 3 was the anaesthesiologist who recorded the findings of the study.

Two manoeuvres were applied by observer 2 to assist in Trachlight™ guided orotracheal intubation. Jaw lift – the non-dominant hand opened the mouth withthe thumb placed against the mandibular incisors while the opposingindex finger was pressed against the ramus of the mandible and a firm anterocaudadforce was applied to provide jaw lift in such a way that the lower incisors were placed anterior to the upper incisors. Combined jaw and tongue traction – after opening the mouth, the tongue was pulled gently out of the oral cavity with the thumb and the index finger of the dominant hand and then the thumb of the non-dominant hand was used to grip the mid portion of the tongue against the mandibular incisors, while the opposingindex finger was pressed against the ramus of the mandible. Then a firm anterocaudadforce was applied to provide combined jaw and tongue traction in such a way that the lower incisors were brought anterior to the upper incisors.

This study was done in two phases: first phase – based on a random pick of lots, one of the airway manoeuvres was applied to aid in Trachlight™ guided intubation. Second phase– the other manoeuvre was applied to aid in Trachlight™ guided intubation.

All patients received 4 μg/kg glycopyrrolate intravenously and two drops oxymetazoline (0.025%) to each nostril half hour prior to surgery. Patients were placed supine on the operating table without a pillow or head ring under the occiput. Standard monitors including the electrocardiogram (lead II), non-invasive blood pressure, pulse oximeter and a peripheral nerve stimulator were established.

Endotracheal tubes of 7.0 and 8.0 mm internal diameter were used for women and men respectively. After preloading the endotracheal tube over the Trachlight™, the Trachlight™-endotracheal tube complex (TEC) was bent into a *‘hockey stick’* configuration at the level of the ‘bend here’ mark on the Trachlight™.

After induction of anaesthesia with 2 mg/kg propofol, 2 μg/kg fentanyl citrate and confirmation of ability to mask ventilate, neuromuscular blockade was achieved with 0.1 mg/kg of vecuronium. Patients were then ventilated through a circle system using bag-mask with 5 l/min oxygen and isoflurane until achieving a zero train-of-four count and an end-tidal isoflurane 1.5 %.

A fibreoptic bronchoscope was advanced by observer 1 into the nasopharynx until its tip was in the oropharynx. At this juncture, the fibrescope was withdrawn till the tip was just at the uvula. View of the glottis at this stage with the head in neutral position was noted. From here on observer 1 continued to visualise the glottic structures (with tip of the fibrescope at uvula) until the completion of intubation. A screen was placed between observers in such a way that observer 2 was blinded to the fibreoptic monitor view and observers 1 and 3 were blinded to the airway manoeuvre. Based on a random pick of lots, observer 2 applied one of the two airway manoeuvres in the first phase of the study followed by the other manoeuvre in the second phase. Trachlight™ guided orotracheal intubation was attempted by observer 2 while sustaining the applied airway manoeuvre throughout the process of intubation. The TEC was advanced into the oral cavity by observer 2 until a well circumscribed midline glow was obtained. The TEC was then advanced gently into the trachea. Observer 1 and 3 noted any laryngeal interference by constantly observing the fibreoptic monitor. Time to intubate was defined as the time from insertion of the TEC into the oral cavity till the glow at the suprasternal notch disappeared (this was conveyed by observer 2). At the end of intubation, fibreoptic assessment for the final position of the epiglottis and any evidence of trauma was noted. Intubation was also confirmed by fibreoptic visualisation of the tube passing between the vocal cords and by capnogram. Following intubation, the patient was ventilated with oxygen and isoflurane till the end tidal isoflurane was 1.5. At this juncture, the trachea was extubated and the second phase of the study was performed with the second manoeuvre after confirming a zero train-of-four count. Same tracheal tube was used with lightwand for second intubation.

During the study, laryngeal interference was assessed independently by both observer 1 (objectively based on the number of times laryngeal interference was observed) and observer 2 (subjectively based on the difficulty encountered while attempting intubation) and graded as nil/minimal, moderate or severe interference [Table 1].

**Table 1:** Subjective and objective evaluation score for laryngeal interference

Grade of laryngeal interference	Observer 2 (subjective evaluation)	Observer 1 (objective evaluation)
Nil/minimal	Nil or minimal difficulty	Nil or minimal interference
Moderate	Some resistance during intubation	<Three contacts of TEC with glottic structures
Severe	Significant resistance during intubation	≥Three contacts of TEC with glottic structures

An oesophageal intubation or failure to intubate within 30 s was regarded as a failed attempt. One attempt was allowed with each manoeuvre. In the case of failure to intubate with the Trachlight™ using the allotted manoeuvre in the second phase of the study, patients would be intubated with any other device deemed appropriate at that juncture.

The grading of fibreoptic view was as follows:

Grade 1: Epiglottis and full view of glottis seen.

Grade 2: Epiglottis and only partial view of the glottis seen.

Grade 3: Epiglottis seen but no glottic structure seen.

Grade 4: Neither epiglottis nor glottic structures seen.

All patients were evaluated for sore throat in the postoperative period prior to discharge from the recovery room. The total duration of surgery was noted and the endotracheal tube cuff pressure monitored at half hourly intervals to keep it below 25 cm H _2_O throughout the duration of surgery.

### Statistical analysis

A comprehensive literature search failed to reveal studies comparing techniques designed to aid difficult lightwand-guided intubation. Therefore, we did a pilot study involving 10 patients. Epiglottic distortion was observed on four occasions, thrice with combined manoeuvre and once with jaw lift manoeuvre. Based on this, we needed to include 76 patients to achieve a power of 80% for our study. We included 84 patients in our study to accommodate for failures. A *P* value of <0.05 was taken as statistically significant.

## RESULTS

**Figure 1:**
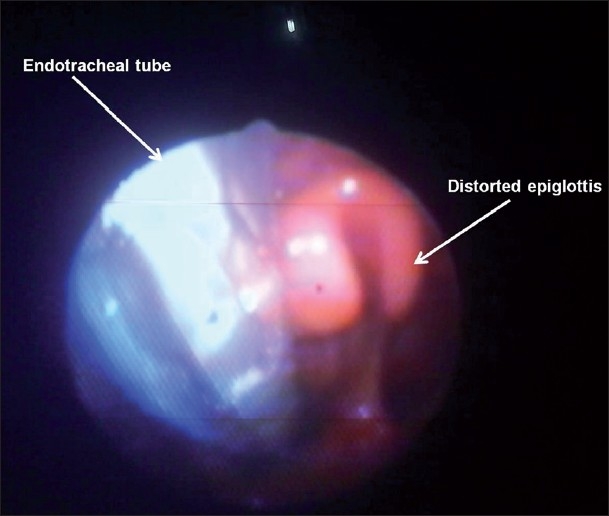
Epiglottis deviated completely to one side of the endotracheal tube

The demographic data is given in Table 2. In 79 patients, fibreoptic visualisation failed to reveal either epiglottis or glottic structures prior to application of airway manoeuvre. A full view of glottis was available in 83 and 80 patients after optimal application of jaw lift and combined maneouvers, respectively. The epiglottis was completely deviated to one side of the endotracheal tube [Figure 1] at the end of intubation on five occasions with jaw lift and on 15 occasions with the combined manoeuver. Infolded epiglottis was observed on one occasion with jaw lift and on two occasions with combined manoeuvre. The total incidence of epiglottic distortion was statistically significant between the two manoeuvres (6 with jaw lift and 17 with combined manoeuvre, *P* = 0.003 [Table 3].

**Table 2:** Demographic data

Parameters	*n* = 84
Male:female	36:48
Age (years)	17 →85^a^
BMI (kg/m^2^)	24.08 (2.08)^b^

a Range

b Mean(± SD).

**Table 3:** Position of the epiglottis at the end of Trachlight™ guided intubation

Manoeuvre	Position			*P* value
	Midline	Deviated	Infolded	
Jaw lift	70	5	1	0.003*
Combined tongue with jaw lift	59	15	2	

McNemar test

* *P* value <0.05, statistically significant.

Objective assessment revealed 39 instances of moderate to severe laryngeal interference: 9 (jaw lift) and 30 (combined manoeuvre) [Table 4], *P* = 0.012. However, subjective assessment revealed 10 (jaw lift) and 12 (combined manoeuvre) instances of moderate to severe laryngeal interference was encountered [Table 4],*P* = 0.832. Perception of laryngeal interference both objectively and subjectively was similar only in six instances with jaw lift and nine instances with the combined manoeuvre.

**Table 4:** Laryngeal interference score

Manoeuvre	Laryngeal interference			*P* value
	Nil / minimal	Moderate	Severe	
Objective				
Jaw lift	72	7	2	0.012*
Combined	48	29	1	
tongue with jaw lift				
Subjective				
Jaw lift	71	9	1	0.832#
Combined tongue with jaw lift	66	11	1	

McNemar test

* *P* value <0.05, statistically significant.

# *P* value <0.05, statistically not significant.

There were nine instances of failure to intubate with Trachlight™ out of 168 attempts (3 with jaw lift and 6 with combined manoeuvre). In all these instances, the attempt to intubate was aborted as time exceeded 30 s (indicated by the blinking of Trachlight™ bulb). Fibreoptic evaluation in these patients revealed that impingement of the TEC on the epiglottis was the main reason for failure to advance despite obtaining a midline glow in all. Trachlight™ guided intubation could not be done with either manoeuvres in one patient who was subsequently intubated with direct laryngoscopy.

Time to intubate was slightly longer with combined manoeuvre as compared to jaw lift (14 vs 11 s, *P*=0.001, Table 5). Two patients each developed mild and moderate sore throat postoperatively. None of the patients had any evidence of direct trauma to any of the oropharyngeal structures. No patient desaturated to <95% SpO_2_ during the study as the time for intubation was limited to <30 s. Hence, no patient required additional oxygen supplementation during intubation attempts.

**Table 5:** Time taken to intubate

Time taken for insertion (seconds)	Jaw lift	Combined tongue with jaw lift	*P* value
Median	11	14	0.001*
25^th^	8	11	
75^th^	14	16	

Wilcoxon-signed rank test

* *P* value <0.05, statistically significant.

## DISCUSSION

Induction of general anaesthesia results in varying degrees of upper airway obstruction ranging from upper airway narrowing to complete airway obstruction in an unprotected airway that may hinder manoeuvrability of lightwand. Several simple manoeuvres may help to overcome this such as jaw lift; use of the thumb of the non-dominant hand to lift the tongue; hyperextension of the head and neck; and having an assistant to pull the tongue forward.[2] A study in children has revealed that jaw thrust often results in lifting the epiglottis off the posterior pharyngeal wall.[7] However, there have been no controlled trials comparing the relative effectiveness of jaw lift versus combined jaw and tongue traction in assisting lightwand-guided intubation. Trachlight™ guided intubation has been found to be difficult in patients with thick, short neck and also in obese individuals mainly in view of increased fat in the neck that masks the light.[8] Several simple steps can help improve the success in these situations such as switching off or dimming the operating room lights and placing a support under the shoulder to extend the neck. Recently, Trachlight™ has also been found to be effective in aiding both open and percutaneous tracheostomy especially in individuals with short, thick necks.[9,10] The device has also been found to be useful in patients with significant maxillofacial trauma.[11] Lightwand has also been included in the difficult airway algorithm limb of American Society of Anesthesiologists.[12] Although tongue traction manoeuvre has been found to be as effective as the jaw lift manoeuvre,[13] we studied the jaw lift manoeuvre versus combined tongue and jaw lift manoeuvre as one of the studies has found that the oropharyngeal clearance was better with the combined manoeuvre for fibreoptic guided intubation.[14]

Fibreoptic evaluation of the airway with the head in neutral position in our study revealed that a majority (79/84) of the patients had complete airway obstruction following anaesthesia and neuromuscular blockade. A full view of glottis was available in 83 and 80 instances after optimal application of jaw lift and combined manoeuvres respectively which is comparable with existing literature evidence.[2,7]

Lightwand-guided intubation is routinely performed by a single individual. Also, prior to commencing the study, our attempts to have the combined manoeuvre executed by two persons with tongue traction provided by one person and jaw lift by the intubator resulted in complexity and space constraints. At the same time, it is not possible to practice all routine lightwand-guided intubations using two operators for performing a combined manoeuvre. Hence, we opted for a single person performing both the manoeuvres.

In our study, the combined manoeuvre had significantly higher laryngeal interference and epiglottic distortion compared to jaw lift (*P* = 0.003, [Table 3]. This may be attributable to the combined manoeuvre being performed by a single individual. Firm pressure on the tongue while attempting intubation might have inadvertently resulted in increased bulk of the base of the tongue. This could have limited the oropharyngeal space available for manoeuvring the TEC. This explains increased incidence of laryngeal interference with combined manoeuvre despite obtaining a full glottic view. The differences in subjective and objective evaluation for laryngeal interference during lightwand-guided intubation [Table 4] confirms earlier findings that the individual performing intubation may not always appreciate interference of the TEC with laryngeal structures.[3] Out of the total 168 attempts at lightwand intubation (84×2 as patients served as their own controls), there were nine failures with overall success rate of >90%. In one lady, intubation was not possible using either manoeuvres who was later intubated with direct laryngoscopy. She possibly had some degree of subglottic stenosis that prevented a 7.0 size tube from entering the trachea and was successfully intubated with a 6.5 size tube. In all others, the cause for failure was mainly due to epiglottic interference with the TEC. Five of them had long and overhanging epiglottis. This confirms previous literature evidence that large epiglottis is one of the reasons for failure to intubate.[2]

Deviated epiglottis was restored to midline in all instances simply by deflating the cuff and rotating the endotracheal tube in the direction of the epiglottis. However, in cases of infolded epiglottis, the endotracheal tube had to be removed in order to restore the epiglottis to original position. As the distortion of epiglottis was corrected immediately after it was noticed, none of the patients had any postoperative complication attributable to the intubation process except mild to moderate sore throat in four patients, which was self-limiting.

The mean time taken to intubate was 11 s with jaw lift manoeuvre and 14 s with combined manoeuvre (*P*=0.001, [Table 5]. However, this difference is clinically irrelevant and the time taken to intubate correlates with existing literature evidence.[2]

There are several limitations to our study. We have not studied the position of laryngeal cartilages in our study. This was mainly due to the absence of recording facility in our fibreoptic monitor. Therefore, the study had to be done in real time and hence, we studied only the position of the epiglottis at the end of intubation. Another important limitation of this study was inability to quantify accurately the volume of the orolaryngeal space.

## CONCLUSION

The results of our study re-emphasise the fact that Trachlight™ guided intubation is a semi-blind technique and the possibility of epiglottic distortion during the process of intubation should be kept in mind. Jaw lift manoeuvre results in less laryngeal interference and epiglottic distortion than combined jaw and tongue traction when applied simultaneously by the non-dominant hand of the person performing intubation.

## References

[1] Hung OR, Stewart RD (1995). Lightwand intubation: I.A new intubating device. Can J Anaesth.

[2] Hung OR, Pytka S, Morris I, Murphy M, Launcelott G, Stevens S (1995). Clinical trial of a new lightwand (Trachlight^®^) to intubate the trachea. Anesthesiology.

[3] Aoyama K, Takenaka I, Nagaoka E, Kadoya T, Sata T, Shigematsu A (2001). Potential damage to the larynx associated with light-guided intubation: A case and series of fiberoptic examinations. Anesthesiology.

[4] Takenaka I, Aoyama K, Nagaoka E, Seto A, Niijima K, Kadoya T (1999). Malposition of the epiglottis after tracheal intubation via the intubating laryngeal mask. Br J Anaesth.

[5] Takenaka I, Aoyoma K, Abe Y, Iwagaki T, Takenaka Y, Kadoya T (2009). Malposition of the epiglottis associated with fibreoptic intubation. J Clin Anaesth.

[6] Debo RF, Colonna D, Dewerd G, Gonzalez C (1989). Cricoarytenoid subluxation: Complication of blind intubation with a lighted stylet. Ear Nose Throat J.

[7] Roth B, Magnusson J, Johansson I, Holmberg S, Westrin P (1998). Jaw lift - a simple and effective method to open the airway in children. Resuscitation.

[8] Umesh G, Mathew G, Ramkumar V (2007). Trachlight® – more practical solutions to commonly encountered problems. Can J Anaesth.

[9] Goneppanavar U, Rao S, Shetty N, Manjunath P, Anjilivelil DT, Iyer SS (2010). Light at a tunnel’s end: The lightwand as a rapid tracheal location aid when encountering false passage during tracheostomy. Indian J Crit Care Med.

[10] Addas BM, Howes WJ, Hung OR (2000). Light-guided tracheal puncture for percutaneous tracheostomy. Can J Anaesth.

[11] Jain S, Bhadani U (2011). Lightwand: A useful aid in faciomaxillary trauma. J Anaesth.

[12] (2003). Practice guidelines for management of the difficult airway: An updated report by the American Society of Anesthesiologists Task Force on Management of the Difficult Airway. Anesthesiology.

[13] Umesh G, George M, Venkateswaran R (2010). Tongue traction is as effective as jaw lift maneuver for Trachlight-guided orotracheal intubation. Acta Anaesthesiol Taiwan.

[14] Durga VK, Millns JP, Smith JE (2001). Maneuvers used to clear the airway during fibreoptic intubation. Br J Anaesth.

